# Lactobacilli and Bifidobacteria Promote Immune Homeostasis by Modulating Innate Immune Responses to Human Rotavirus in Neonatal Gnotobiotic Pigs

**DOI:** 10.1371/journal.pone.0076962

**Published:** 2013-10-02

**Authors:** Anastasia N. Vlasova, Kuldeep S. Chattha, Sukumar Kandasamy, Zhe Liu, Malak Esseili, Lulu Shao, Gireesh Rajashekara, Linda J. Saif

**Affiliations:** The Food Animal Health Research Program, Ohio Agricultural Research and Development Center, Veterinary Preventive Medicine Department, The Ohio State University, Wooster, Ohio, United States of America; Virginia Polytechnic Institute and State University, United States of America

## Abstract

The effects of co-colonization with *Lactobacillus rhamnosus* GG (LGG) and *Bifidobacterium lactis* Bb12 (Bb12) on 3-dose vaccination with attenuated HRV and challenge with virulent human rotavirus (VirHRV) were assessed in 4 groups of gnotobiotic (Gn) pigs: Pro+Vac (probiotic-colonized/vaccinated), Vac (vaccinated), Pro (probiotic-colonized, non-vaccinated) and Control (non-colonized, non-vaccinated). Subsets of pigs were euthanized pre- [post-challenge day (PCD) 0] and post (PCD7)-VirHRV challenge to assess diarrhea, fecal HRV shedding and dendritic cell/innate immune responses. Post-challenge, Pro+Vac and Vac groups were completely protected from diarrhea; protection rates against HRV shedding were 100% and 83%, respectively. Diarrhea and HRV shedding were reduced in Pro compared to Control pigs following VirHRV challenge. Diarrhea scores and virus shedding were significantly higher in Controls, compared to all other groups, coincident with significantly higher serum interferon-alpha levels post-challenge. LGG+Bb12 colonization ±vaccine promoted immunomaturation as reflected by increased frequencies of CD4, SWC3a, CD11R1, MHCII expressing mononuclear cells (MNCs) and conventional dendritic cells in intestinal tissues and blood post-challenge. Colonization decreased frequencies of toll-like receptors (TLR) 2 and TLR4 expressing MNCs from vaccinated pigs (Pro+Vac) pre-challenge and increased frequencies of TLR3 expressing MNCs from Pro pigs post-challenge, suggesting that probiotics likely exert anti-inflammatory (TLR2 and 4 down-regulation) and antiviral (TLR3 up-regulation by HRV dsRNA) actions via TLR signaling. Probiotic colonization alone (Pro) increased frequencies of intestinal and systemic apoptotic MNCs pre-challenge, thereby regulating immune hyperreactivity and tolerance. However, these frequencies were decreased in intestinal and systemic tissues post-challenge, moderating HRV-induced apoptosis. Additionally, post-challenge, Pro+Vac and Pro groups had significantly decreased MNC proliferation, suggesting that probiotics control excessive lymphoproliferative reactions upon VirHRV challenge. We conclude that in the neonatal Gn pig disease model, selected probiotics contribute to immunomaturation, regulate immune homeostasis and modulate vaccine and virulent HRV effects, thereby moderating HRV diarrhea.

## Introduction

The human gastrointestinal tract, inhabited by over 1,000 species of microorganisms, has a very high level of metabolic and immune activities [1] and sustains the balance between immunity and tolerance. After birth, mucosal surfaces (including gut) are colonized by a complex succession of different bacteria that direct morphologic and functional maturation of the neonatal immune system [2]. The type of feeding, mode of delivery and the gestational age shape the composition of intestinal microflora [2,3]. Deviant or significantly altered (by antibiotic overuse) gut microbiota can predispose to numerous metabolic (autoimmune and inflammatory) conditions and infectious diseases [4,5].

Most of the current knowledge of microbiota–host interactions has been acquired using gnotobiotic (Gn) animals that can be selectively colonized with defined microflora. Using Gn mice it was first shown that commensal bacteria stimulate proliferation of IgA plasma cells and overall development of the intestinal immune system during neonatal life [6,7]. Colonization of Gn mice by segmented filamentous bacteria resulted in increased constitutive cytotoxicity of CD8+ T cells and decreased frequencies of unprimed T cells, suggesting activation of the cellular compartment of the immune system [6,8]. Butler et al. using Gn pigs further demonstrated stimulatory effects of commensal microflora on differentiation of naive B cells into plasma cells and/or expression of co-stimulatory molecules (for T and B cells) by antigen presenting cells [9]. They also showed that the commensal microflora stimulates antibody production to non-bacterial T cell dependent and independent antigens and plays a major role in the overall development of the systemic and mucosal immune systems of the neonate [9]. Interestingly, in our earlier studies using Gn pigs, we demonstrated that virulent human rotavirus (VirHRV) infection alone was as efficient at promoting intestinal B cell responses as co-colonization with 2 strains of lactobacilli [10]. Recently, a marked effect of microbial colonization on the mRNA expression of intestinal chemokines, chemokine receptors, Foxp3 and TGF-β was demonstrated using the Gn pig model [11]. Gram-positive [*Lactobacillus fermentum* (*L. fermentum*)] and gram-negative (*E. coli*) bacterial species were shown to differentially affect small intestine morphology and inflammatory cytokine profiles in Gn pigs [12]. Additionally, Shirkey et al. demonstrated that non-colonized/monocolonized Gn pigs exhibited extreme vacuolization of enterocytes along the villi of the distal small intestine, reduced relative small intestinal length and decreased numbers of intraepithelial lymphocytes as compared to conventionalized pigs [12]. Another study demonstrated that conventional bacteria and *E. coli* but not *L. fermentum* increased overall enterocyte turnover through stimulating apoptosis and cell proliferation [13]. Collectively, these findings using Gn animals support the hypothesis that postnatal bacterial colonization patterns have a profound and multifaceted effect on development of the neonatal immune system.

Rotavirus (RV) is a leading cause of viral diarrhea in infants and children. It is associated with ^≈^453,000 deaths in children younger than 5 years worldwide annually [14]. Two currently licensed live attenuated RV vaccines, although effectively preventing severe RV gastroenteritis in developed countries (>80%), due to unknown reasons showed reduced efficacy (~50%) in impoverished countries, where RV diarrhea is most severe [15].

Probiotics are increasingly recognized as alternative low cost treatments to moderate infectious diarrhea and to improve intestinal homeostasis [16,17]. Some lactic acid bacteria (LAB) were shown to significantly stimulate gut epithelial cell proliferation [18], reduce gut permeability, enhance immune responses and provide other health benefits [19,20]. *L. reuteri, L. rhamnosus* and *L. acidophilus* reportedly reduced the duration of RV diarrhea or shedding in children [21-23]. The beneficial effects observed in these studies appear to be bacteria and host species-specific and the precise molecular mechanisms of the underlying host-microbe interactions remain largely unknown. However, recent data suggest that probiotics promote intestinal health rather through stimulation (not suppression) of innate immunity [24] and that dendritic cells (DCs) may play a key role in probiotic functionality [25].

The neonatal Gn pig model of human rotavirus (HRV) infection and disease is a highly relevant model to study HRV pathogenesis, protective immunity to HRV infection and vaccines [26]. Because neonatal Gn pigs resemble human infants in gastrointestinal physiology and development of immune responses and are devoid of normal microflora, they are a unique animal model to investigate initial interactions between the microflora, the immune system of neonates and enteric pathogens (RVs) [27]. Earlier we demonstrated that mono-colonization of Gn pigs with lactobacilli (*L. acidophilus*) enhances the immunogenicity of the oral attenuated HRV vaccine [28] and that co-colonization with lactobacilli (*L. acidophilus* and *L. reuteri*) modulates immune responses to virulent HRV, affecting frequencies of B cells, monocytes/macrophages and DCs, toll-like receptors (TLRs) and innate cytokine expression patterns [10,27,29]. However, HRV diarrhea was not alleviated by short-term lactobacilli colonization (only 2 days prior to VirHRV) and HRV vaccine protective efficacy was not assessed in these earlier experiments.

In this study we evaluated the impact of co-colonization by *Lactobacillus rhamnosus* GG (LGG) and *Bifidobacterium lactis* Bb12 (Bb12), two commensals selected because they are dominant in breast-fed infants, on HRV diarrhea, development of innate immune responses and vaccine efficacy using a neonatal Gn pig model of HRV vaccination and infection.

## Materials and Methods

### Virus

The virulent HRV (VirHRV) Wa strain was used for inoculation at a dose of 1x10^5^ fluorescent-forming units (FFU). The 50% infectious dose (ID_50_) of VirWaHRV in pigs was determined as approximately 1 FFU [30]. Cell-culture adapted attenuated Wa strain HRV (AttHRV) was maintained in MA 104 cells and used as the monovalent attenuated HRV vaccine [3 doses of 5 × 10^7^ fluorescent-forming units (FFU)/dose] and positive control in cell culture immunofluorescence assay (CCIF) [30].

### Bacterial strains

The probiotics *Lactobacilli rhamnosus GG* (LGG) strain ATCC 53103 (ATCC, Manassas, VA, USA) and *Bifidobacterium animalis* *subsp.* *lactis* Bb12 (Christian Hansen Ltd., Hørsholm, Denmark) were used to colonize Gn pigs. The LGG and Bb12 were propagated and enumerated as described previously [31].

### Ethics statement

This study was carried out in strict accordance with the recommendations by Public Health Service Policy, United States Department of Agriculture Regulations, the National Research Council’s Guide for the Care and Use of Laboratory Animals, and the Federation of Animal Science Societies’ Guide for the Care and Use of Agricultural Animals in Agricultural Research and Teaching, and all relevant institutional, state, and federal regulations and policies regarding animal care and use at The Ohio State University. The protocol (#2010A00000088) was approved by the Committee on the Ethics of Animal Experiments of The Ohio State University. All the pigs were maintained, samples collected, and euthanized, and all efforts were made to minimize suffering of animals. Oral inoculation of neonatal pigs with Wa RVA caused transient diarrhea of variable severity from which piglets recovered spontaneously within a few days. The euthanasia was performed by electrocution following anesthesia. For this particular study, we used the lowest number of pigs previously shown to permit detection of statistical significances among treatments. This minimum number of pigs required per treatment group was verified by the statistician before starting the experiments.

### Experimental design

Gnotobiotic pigs from three sows were derived near term and maintained in sterile isolation units as described previously [32]. All the pigs were fed with ultra-high-temperature processed commercial milk throughout the experiment. To assess the effects of probiotic colonization on the immune responses to and protective efficacy of AttHRV Wa, pigs from 3 litters were assigned to one of 4 treatment groups as follows: 1) 3xAttHRV Wa vaccinated, LGG+Bb12 colonized (Pro+Vac); 2) 3xAttHRV Wa vaccinated, non-colonized (Vac); 3) non-vaccinated, LGG+Bb12 colonized (Pro); 4) non-vaccinated, non-colonized controls (Control). Pro+Vac and Vac pigs were vaccinated as described above. Gn pigs (in the probiotic colonized groups) were colonized with selected probiotic strains, LGG and Bb12, sequentially as follows: first, pigs were inoculated with only Bb12 at a dose of 1×10^5^ CFU at 3 days of age to establish its colonization prior to the LGG dose. Subsequently, the Bb12 colonized pigs were inoculated with both LGG and Bb12 at 1:1 ratio (2×10^5^ CFU of each/pig) at 5 days of age. Three sequential vaccinations with oral AttHRV [post-inoculation day 0 (PID0), PID10 and PID21] were done 10 days apart starting at 6-days of age (PID0). Seven days after the last vaccination, all pigs were challenged with homologous VirHRV Wa [PID28/post-challenge day (PCD) 0]. Prior to each AttHRV/VirHRV inoculation, pigs received 5 ml of 100mM NaHCO3 to reduce gastric acidity. Rectal swabs were collected and diarrhea was assessed daily through PCD7 (end of the experiment). Pigs were examined daily for diarrhea post-challenge as described previously [10]. Serum samples were collected at PID 0, 2, 4, 28/PCD0, PID30/PCD2, PID32/PCD4 and PID35/PCD7 to assess IFNα levels. Subsets of pigs were euthanized at PID28/PCD0 and PID35/PCD7 to isolate mononuclear cells (MNC) from ileum, duodenum, spleen and peripheral blood to assess frequencies of DCs, TLR2,3,4 and 9 expressing MNCs, apoptotic MNCs, MNC proliferation etc.

### Fecal shedding and intestinal tissue distribution of probiotic bacteria

Rectal swabs were collected at weekly interval from all pigs for enumeration of fecal bacterial shedding in probiotic colonized pigs and for sterility assessment in non-colonized pigs. Intestinal bacterial colonization was assessed by enumerating CFUs per gram of homogenized tissue sections collected from duodenum, jejunum, ileum, cecum and colon as described [10]. Total probiotic (LGG+Bb12) counts in the gut tissues, including bacteria in the mucus layer, epithelial surface and in the tissue, are referred to as mucosa associated bacteria. The specific colonization of LGG and Bb12 was determined by quantitative polymerase chain reaction (qPCR) using DNA extracted from representative rectal swab fluids and tissues using LGG and Bb12 specific primers and probes (LGG primers/probe; courtesy of Dr. Gloria Solano-Aguilar, USDA, and Bb12 [33]).

### Detection of rectal HRV shedding by CCIF

Rectal swab samples were tested by CCIF to quantitate infectious HRV, as previously described [30].

### Isolation of mononuclear cells (MNCs)

Spleen, blood, duodenum and ileum were collected the day of euthanasia and processed for isolation of MNC as previously described [34]. Flow cytometry staining was performed the same day immediately after the isolation of all tissue-derived cells.

### Flow cytometry staining

Procedures for flow cytometry staining (including buffers used) were performed as described previously [35]. To assess frequencies and distribution of DC subsets, MHCII expression on MNCs and DCs and TLR expression by MNCs, cells were stained with monoclonal antibodies to porcine and human cell surface markers/TLRs, isotype control and secondary antibodies (see Table S1). Plasmacytoid DCs were defined as SWC3a+CD4+CD11R1-, conventional DCs were defined as SWC3a+CD4-CD11R1+; additionally, expression of CD103 marker (an integrin mediating lymphocyte/DCs retention in mucosal tissue) was assessed for both DC subsets and total MNC population. After the cell surface marker/TLR2/TLR4 stainings were completed, the cells were permeabilized as described previously [35]. Intracellular staining for TLR3 and TLR9 was performed as recommended by the manufacturer. Acquisition of 50,000 events was done using AccuriC6 flow cytometer (Accuri cytometers, Inc.). Analyses were performed using CFlow software (BD, Accuri cytometers, Inc.).

### Frequencies and tissue distribution of apoptotic and necrotic MNC by flow cytometry

Annexin V Apoptosis Detection Kit APC (eBiosciences, San Diego, CA) and Propidium Iodide Staining Solution (eBiosciences) were used according to the manufacturer protocols to detect and discriminate apoptotic and necrotic MNCs. Within 4 hours after the staining, acquisition of 50,000 events was performed using AccuriC6 flow cytometer and analyses were performed using CFlow software.

### Proliferation assay

To assess basal MNC *ex vivo* proliferation we used Click-iT® EdU Alexa Fluor® 488 Flow Cytometry Assay Kit (Invitrogen, Grand Island, NY) according to the manufacturer’s instructions.

***Interferon-αproductionbysplenicMNCsafterinvitrore-stimulationandIFNαbioassay*** were performed as described previously [35].

### In vitro stimulation with LGG, Bb12 and HRV antigen

Freshly isolated MNCs from spleen of 4-week-old control Gn pigs (without previous exposure to any bacteria/viruses) were co-cultured with the live probiotic bacteria (re-suspended in PBS) LGG, Bb12, LGG+Bb12 (at 1:1 ratio) with bacteria:MNC ratio of 10:1, purified inactivated AttHRV (HRV) (12µg/ml) (re-suspended in PBS) or LGG+Bb12+HRV for 48 hrs at 37°C with 5% CO_2_. PBS was included as a control treatment. After 48 hrs, MNCs were washed with cold sterile PBS and subjected to flow cytometry staining (to assess TLR2+, TLR3+, TLR4+, TLR9+ and apoptotic MNC and pDC/MHCII+ pDC and cDC/MHCII+cDC frequencies) and analysis was done as described above. Supernatants were harvested and stored at -80 °C until tested. The TGFβ (Th3), TNFα (pro-inflammatory), IL10 (Th2), and IL12 (Th1) cytokine levels were measured by ELISA as previously described [36]; IFNα (innate) was measured by IFNα bioassay as previously described [35].

### Statistical analyses

One-way analysis of variance (ANOVA-general linear model) was used to compare mean duration of virus shedding/diarrhea, and IFNα levels. The frequencies of cell populations in flow cytometry were compared among or within groups using the Wilcoxon rank-sum test (non-parametric) test. The Spearman rank correlation method was used to estimate the associations between TLR expressing/apoptotic MNC frequencies, VirHRV shedding and serum IFNα levels. Statistical significance was assessed at p ≤0.05 for all comparisons. All statistical analyses were performed using the Minitab 16 program (Minitab Inc., PA, USA) and GraphPad Prism 5 (GraphPad Software, Inc., CA, USA).

## Results

### Total LGG and Bb12 numbers were higher in non-vaccinated pigs

The two bacterial strains colonized Gn pigs effectively. We confirmed the presence of both LGG and Bb12 in rectal swabs and tissues from representative pigs from each group by qPCR using species-specific primers and probes. LGG and Bb12 were detected at similar levels, with LGG counts slightly higher than Bb12 (~1.2 fold in gut tissues and ~1.3 fold in rectal swabs, data not shown).

**Figure 1:**
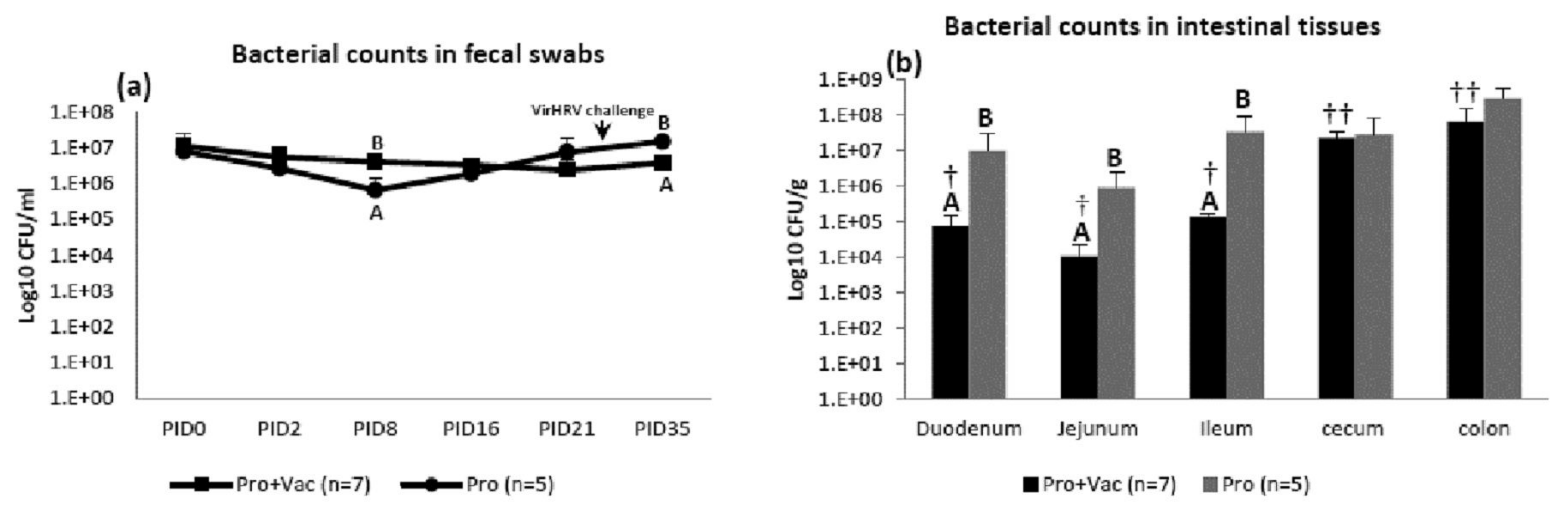
Probiotic (LGG+Bb12) counts in feces and intestinal tissues. (**a**) Total rectal LGG and Bb12 shedding expressed as mean numbers of LGG+Bb12 (CFU/ml) recovered from rectal swabs. Letters A and B indicate significant (p<0.05) difference between total bacterial numbers of Pro+Vac and Pro groups. (**b**) Total numbers and tissue distribution of mucosa associated LGG and Bb12 at PID28/PCD35. Letters A and B indicate significant (p<0.05) difference between total bacterial numbers of Pro+Vac and Pro groups. Symbols † and † † indicate significant (p<0.05) difference between total bacterial numbers in small (duodenum, jejunum and ileum) and large (cecum and colon) intestine of Pro+Vac group.

Initially (at PID0), total fecal bacteria shedding was similar between both colonized groups (Pro+Vac and Pro) but post-vaccination, at PID2 it was lower and at PID8 it was significantly lower in the Pro group. However, fecal bacterial counts in Pro pigs increased by PID16, became higher than in Pro+Vac pigs by PID21 and were significantly higher post-VirHRV challenge at PID35/PCD7 (termination of the experiment) (Figure 1a). Therefore, the dynamic of fecal probiotic shedding differed between the colonized Pro+Vac and Pro groups. Pro+Vac pigs maintained similar fecal bacterial counts throughout the experiment ranging between 1.11E+07 CFU/ml (at PID0) and 3.76E+06 CFU/ml (at PID35) and representing no increase or even a slight decrease in total bacterial counts, whereas Pro pigs had a clear trend for an increase in bacterial counts after PID8: from 6.581E+05 CFU/ml (at PID8) to 1.47E+07 CFU/ml (at PID35/PCD7) (Figure 1a). No effect of VirHRV challenge on bacterial counts was evident for Pro+Vac or Pro groups.

We also assessed mucosa-associated bacterial counts in duodenum, jejunum, ileum, cecum and colon (Figure 1b). At PID35/PCD7 for Pro+Vac pigs, LGG+Bb12 numbers were significantly lower in small intestine (1.09E+04 - 1.39E+05 CFU/g) compared to large intestine (2.39E+07 - 6.63E+07 CFU/g). However, for Pro pigs the difference between bacterial counts in small and large intestine was less and not statistically significant: 9.26E+05 - 3.40E+07 and 2.85E+07 - 3.02E+08 CFU/g, respectively. Additionally, numbers of mucosa-associated bacteria were higher in all gut tissues of Pro pigs. Thus, AttHRV oral vaccination resulted in a significant decrease of total small intestinal bacterial counts. Although no probiotic colonized Gn pigs exhibited diarrhea or other clinical signs, higher fecal bacterial loads in Pro pigs may indicate a less mature state of their immune system in the absence of AttHRV (immunostimulator) [27] that may account for a slight overgrowth of probiotic bacteria. Alternatively, it may be a result of interference between these bacteria and AttHRV in the gut of Pro+Vac Gn pigs.

### Diarrhea and HRV shedding titers were lower in LGG and Bb12 colonized Gn pigs

**Figure 2:**
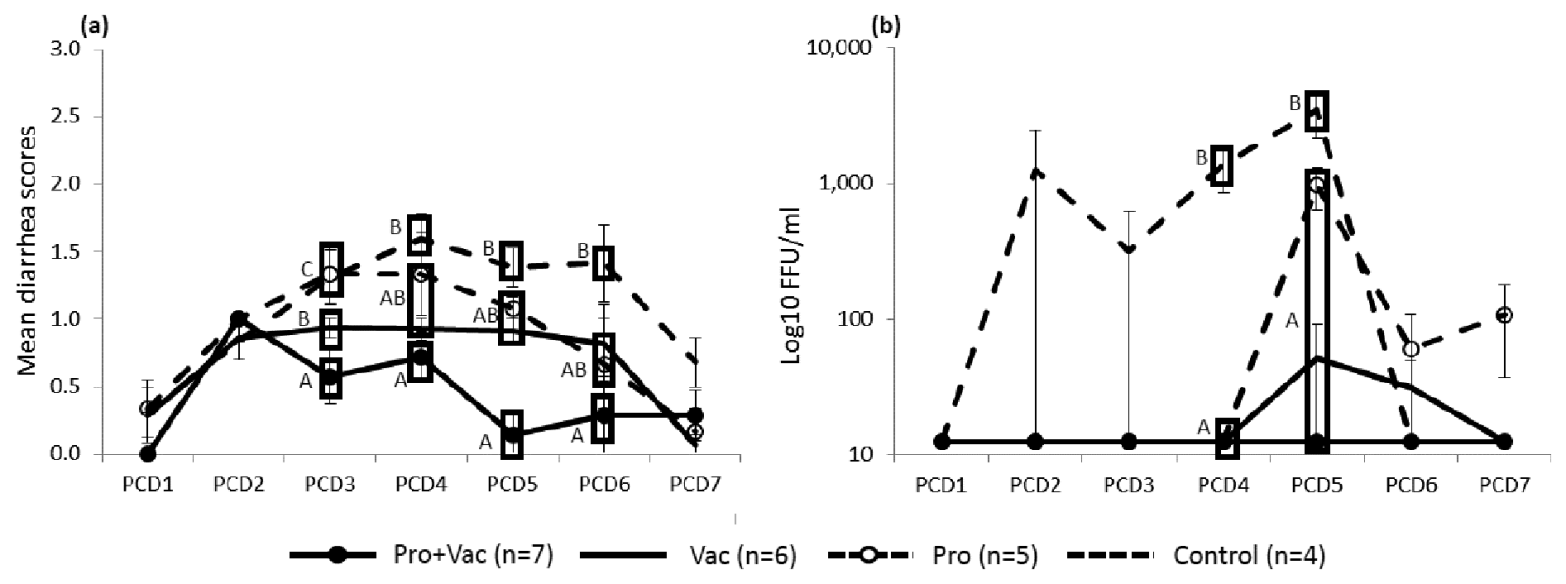
Diarrhea severity and HRV fecal shedding post-VirHRV challenge in vaccinated and control pigs. (**a**) Mean diarrhea scores and (**b**) HRV fecal shedding in different groups. Different letters indicate significant differences (determined by one-way analysis of variance [ANOVA] followed by Duncan’s multiple range test, p<0.05) at the same time-point among treatment groups.

After VirHRV challenge, mean fecal scores were significantly lower and virus shedding titers were lower in Pro+Vac pigs compared to the Vac pigs (Figure 2). Control pigs shed VirHRV at significantly higher titers compared to Pro pigs, with two characteristic peaks of viral replication [previously described in mice and pigs [37,38]:] detected at PCD 2 and 5 (Figure 2). We also observed significantly delayed onset (5 vs. 3.5 days) of HRV shedding with significantly lower virus titers (977.5 vs. 3343.75 FFU/ml), significantly reduced diarrhea duration (1.2 vs. 3 days) and significantly lower cumulative diarrheal scores (5.7 vs. 9) in Pro compared to Control pigs [39]. Protection rates against diarrhea were higher in Pro (20% protection) compared to Control (0% protection) pigs post-VirHRV challenge. Likewise Pro+Vac pigs had higher protection rates against shedding than Vac pigs: 100% vs. 83%, respectively [39].

### LGG and Bb12 colonization affected MNC surface phenotype and dendritic cell (DC) frequencies and distribution post-challenge

**Figure 3:**
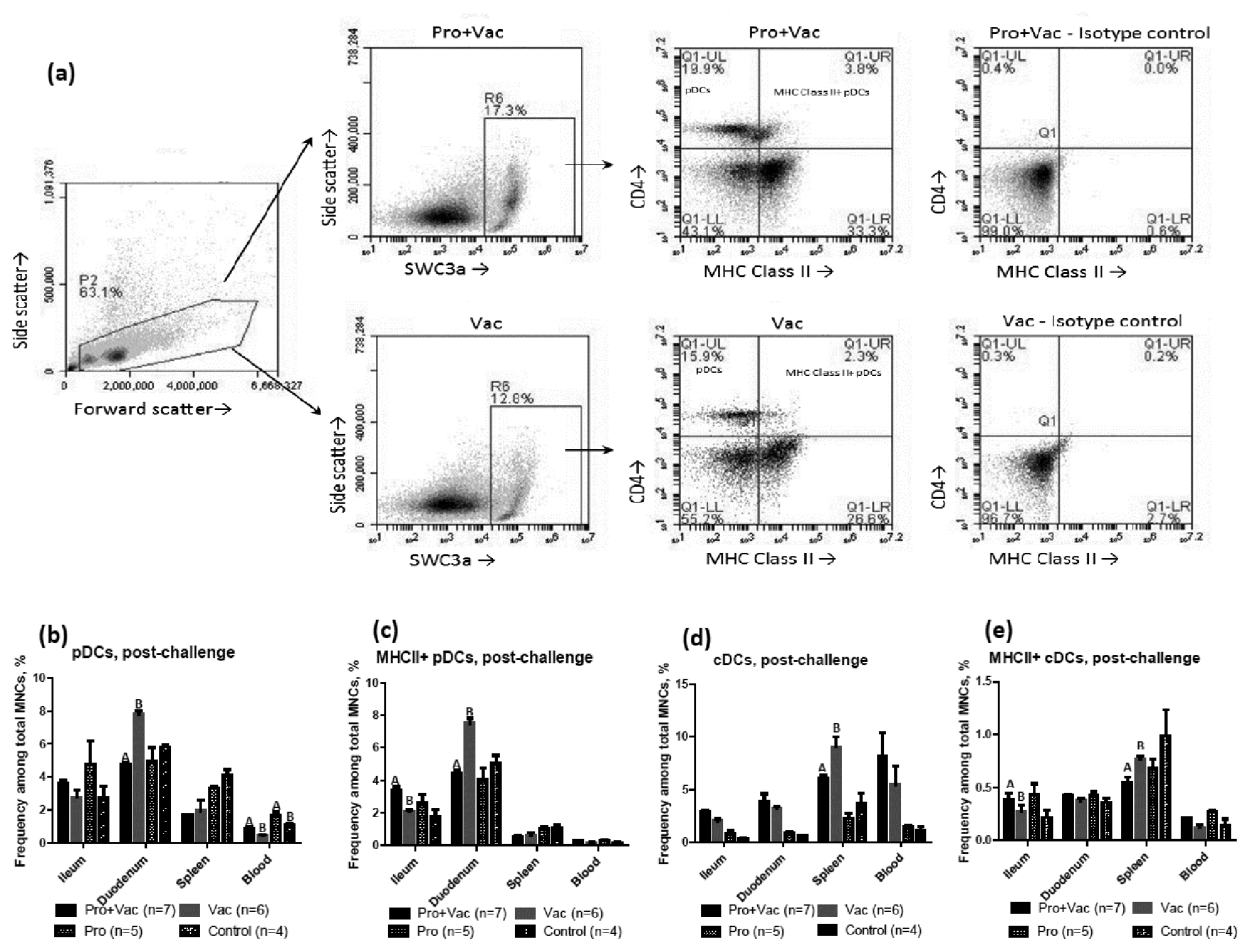
LGG+Bb12 colonization effect on frequencies and tissue distribution of conventional and plasmacytoid dendritic cells. Representative dot plot (**a**) demonstrates sequential gating strategy for MHC II+ pDCs among total MNCs (for spleen samples from Pro+Vac and Vac pigs, post-challenge). Total frequencies of (**b**) pDCs and (**d**) cDCs and frequencies of (**c**) MHC II+ pDCs and (**e**) cDCs in gut (duodenum and ileum) and systemic tissues (spleen and blood) of Gn pigs post-challenge. Letters A and B indicate significant (p<0.05) difference between total bacterial numbers of Pro+Vac and Vac or Pro and Control groups.

The immunomodulatory effects of LGG+Bb12 colonization were evident in Gn pigs only post-challenge. Consistent with the observations for mice, we demonstrated that LGG+Bb12 colonization increased frequencies of CD4, SWC3a, CD11R1 and MHC II expressing MNCs isolated from ileum, duodenum (except pDCs) and blood of probiotic colonized pigs (Pro+Vac and Pro) compared to non-colonized (Vac and Control) pigs post-challenge (Table 1). Similarly, pDC/cDC and MHCII+ pDC/cDC frequencies were also increased in ileum and blood of colonized pigs post-challenge (Table 1; Figure 3). In duodenum frequencies of cDCs (but not pDCs) and MHCII+ cDCs (but not MHCII+ pDCs) were also increased in Pro+Vac and Pro pigs compared to Vac and Control pigs, respectively, but to a lesser extent than in ileum and blood (Table 1; Figure 3). Interestingly, CD4, SWC3a, CD11R1 and MHCII marker expression on MNCs or DC frequencies were mostly either decreased or unaltered for MNCs isolated from spleen of Pro+Vac and Pro vs. Vac and Control pigs (Table 1; Figure 3) that likely indicates systemic regulatory effects of probiotic colonization. Coincidentally, duodenum and spleen are major residential tissues for pDCs and cDCs in the Gn pig model, respectively (data not shown). Although some probiotics were shown to exert their immunomodulatory effects via CD103+ DCs, colonization or vaccination did not affect CD103 expressing MNC or DC frequencies pre- or post-challenge in this study (data not shown).

**Table 1:** Effect of probiotic colonization [fold change data (a)] on mononuclear cell (MNC) surface phenotypes, dendritic cell (DC) frequencies and tissue distribution assessed by flow cytometry post-challenge at PID35/PCD7.

	**MHC II+ MNCs**	**SWC3a+ MNCs**	**CD4+ MNCs**	**CD11R1+ MNCs**	**pDCs**	**MHC II+ pDCs**		**cDCs**	**MHC II+ cDCs**
				**Ileum**					
**Pro+Vac/Vac**	1.2	**1.53**	1.64	**2.03**	1.61	**1.32**	**1.45**		1.4
**Pro/Control**	1.14	1.6	**1.79**	**2.15**	1.71	1.47	2.56		1.51
				**Duodenum**					
**Pro+Vac/Vac**	1.2	1.12	1.3	1.3	**0.6**	**0.59**	1.23		1.13
**Pro/Control**	1.17	1.06	1.01	1.22	0.85	0.96	1.33		1.03
				**Spleen**					
**Pro+Vac/Vac**	1	0.79	1.19	0.76	0.67	1.1	**0.61**		**0.76**
**Pro/Control**	1.13	0.75	0.73	0.67	**0.75**	0.93	0.62		0.68
				**Blood**					
**Pro+Vac/Vac**	**1.79**	**1.4**	**2.29**	1.34	**1.5**	1.4	1.12		1.33
**Pro/Control**	2.02	**1.38**	**2.04**	1.53	**1.76**	1.9	1.17		1.43

(a) Fold change = value reflecting the average level of increase or decrease in MNC or DC frequencies in probiotic colonized groups compared to non-colonized groups. Values greater or less than 1 correspond to increase (values more than 1) or decrease (values less than 1) of MNC or DC frequencies, respectively. Bolded values reflect significant difference between colonized and non-colonized groups by non-parametric Mann-Whitney test (P<0.05).

### Frequencies of TLR2, TLR4, TLR9 (pre-challenge) and TLR3 (post-challenge) expressing MNCs were differentially affected by LGG and Bb12 colonization

The combined effects of LGG+Bb12 and AttHRV on constitutive expression of TLR2 [ligands – bacterial peptidoglycan (PGN), lipoteichoic acid, and lipoproteins], TLR4 (ligand – bacterial lipopolysaccharide) and TLR9 (ligand – bacterial CpG) by MNCs were only evident pre-challenge; whereas responsive TLR3 (ligand - dsRNA) expression by MNCs was only evident post-challenge and only in the non-vaccinated pigs (developed HRV diarrhea). No dominant cell population expressing TLR2, TLR3, TLR4 or TLR9 was identified; rather TLR expressing MNCs were represented by diverse immune cell populations, including cDCs, pDCs and other subsets (data not shown).

**Figure 4:**
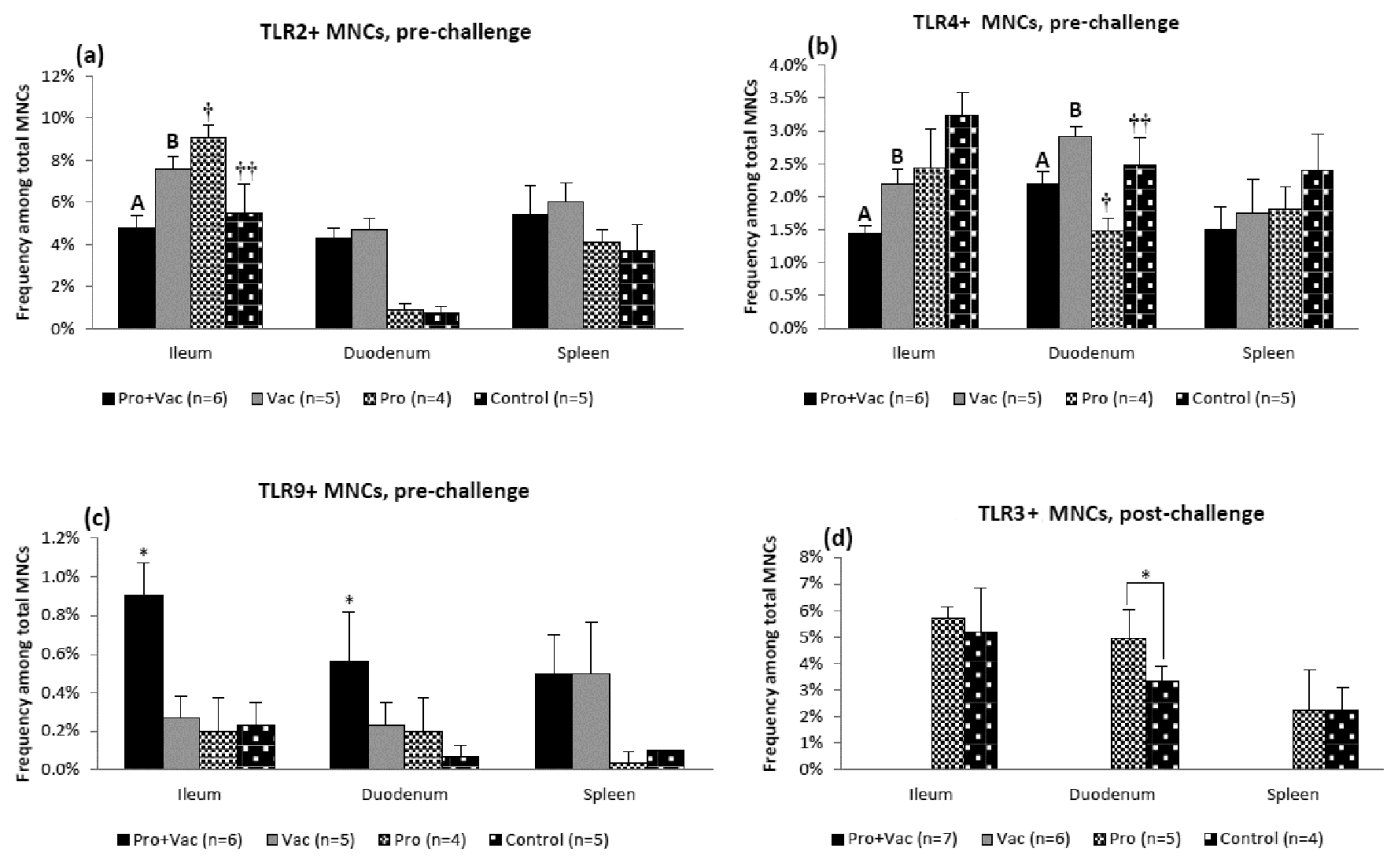
LGG+Bb12 colonization effect on frequencies and tissue distribution of TLR expressing MNCs. (**a**) TLR2, (**b**) TLR4 and (**c**) TLR9 expression pre-challenge and (**d**) TLR3 expression post-challenge by MNCs of Gn pigs. Frequencies of blood MNCs expressing TLRs were very low (<0.4%) and are not shown. Post-challenge TLR3 expression by MNCs from vaccinated pigs (Pro+Vac and Vac) was undetectable. Letters A and B indicate significant (p<0.05) difference between total bacterial numbers of Pro+Vac and Vac groups. Symbols † and † † indicate significant (p<0.05) difference between Pro and Control groups. Asterisk (**c**) indicates that frequencies of TLR9 expressing MNCs were significantly (p<0.05) higher for Pro+Vac compared to Vac, Pro and Control pigs.

Pre-challenge, probiotic colonization of vaccinated pigs significantly decreased frequencies of TLR2 expressing MNCs from ileum and marginally decreased frequencies of TLR2 expressing MNCs from duodenum and spleen from (Pro+Vac vs. Vac) pigs, whereas in unvaccinated control pigs, LGG and Bb12 colonization resulted in increased frequencies of TLR2 expressing MNCs (Pro vs. Control) (Figure 4a). This suggests that in the absence of AttHRV vaccine, the PGN (and other TLR2 ligands) of gram-positive LGG and Bb12 stimulates TLR2 expression, but in vaccinated pigs, LGG and Bb12 components/metabolites antagonize likely AttHRV-induced TLR2 up-regulation [40] presumably through interactions between different microbe associated molecular patterns (MAMPs) and their receptors.

Similarly, frequencies of TLR4 expressing MNCs were also decreased (significantly in intestinal tissues) by probiotic colonization in Pro+Vac vs. Vac pigs. However, unlike for TLR2, they were also decreased (significantly in duodenum) in probiotic colonized (Pro) vs. non-colonized (Control) control pigs (Figure 4b). This is consistent with the fact that gram-positive bacteria lack lipopolysaccharides (LPS) – the TLR4 ligand, but can employ mechanisms counteracting TLR4-mediated pro-inflammatory signaling. No effects of LGG+Bb12 colonization on the frequencies of TLR2 and TLR4 expressing MNCs were observed post-challenge (data not shown).

The total TLR9+ MNC frequencies were low and varied pre-challenge (and were undetectable post-challenge), but in contrast to the frequencies of TLR2/TLR4 expressing MNCs, they were significantly increased in intestinal tissues of Pro+Vac pigs (Figure 4c). This suggests a possible synergistic effect of probiotic colonization and AttHRV vaccine in TLR9 expression up-regulation.

In contrast to the other TLRs, responsive TLR3 (ligand - dsRNA) expression by MNCs (isolated from intestinal and systemic tissues) was only observed post-challenge and only in MNCs from non-vaccinated Pro and Control pigs indicating a specific response to the dsRNA of replicating VirHRV in these challenged susceptible pigs (Figure 4d). The lack of TLR3 expressing MNCs from vaccinated pigs coincides with their protection from VirHRV infection. Interestingly, TLR3 expressing MNC frequencies were significantly increased in duodenum of Pro vs. Control pigs post-challenge (Figure 4d) possibly indicating an immunoactivating role of probiotic colonization. TLR3 expressing MNC frequencies were significantly negatively correlated with VirHRV shedding (R=-0.77; p=0.048) for duodenum but not for ileum and spleen.

Overall, frequencies of TLR2 (0.75-9.06%) and TLR3 (2.2-5.7%) expression were higher than those of TLR4 (1.45-2.45%) (Figure 4a,b,d), which may reflect the presence of specific TLR2 and TLR3 ligands (PGN, lipoproteins, etc. and dsRNA, respectively) and the absence of these for TLR4

(no LPS.) Higher frequencies of TLR2+ and TLR3+ MNCs in ileum as compared to duodenum and spleen (Figure 4a,d) may be related to higher bacterial load and HRV replication primarily in the distal part of the small intestine.

TLR2, TLR3, TLR4 and TLR9 expression levels by MNCs from blood were either undetectable or did not reveal any trends and therefore the data is not shown.

### IFNα responses were higher in sera of non-colonized control pigs post-challenge

**Figure 5:**
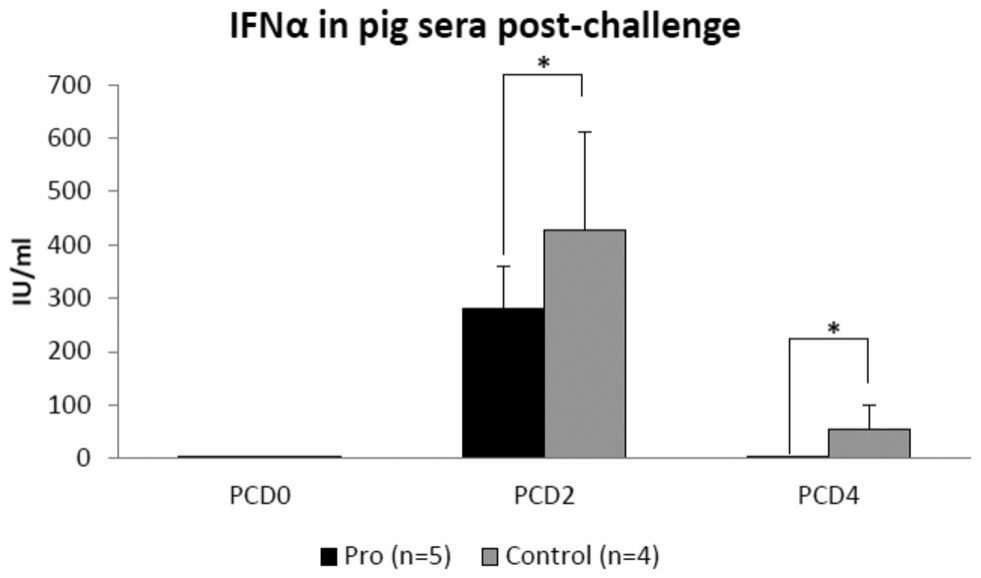
IFNα in pig sera at post-challenge days (PCDs) 0, 2 and 4 assessed by IFNα bioassay. Vaccinated pigs (Pro+Vac and Vac) had undetectable levels of IFNα in sera. Asterisks indicate that non-colonized control pigs had significantly (p<0.05) higher concentrations of serum IFNα as compared to the colonized pigs at PCD2 and 4.

Consistent with higher HRV fecal shedding in Control vs. Pro pigs, we observed higher IFNα levels in serum of Control pigs post-challenge (Figure 5). Furthermore, IFNα was detectable at PCD2 in Pro and Control pigs, while at PCD4 circulating IFNα could only be detected in the more severely affected Control pigs (Figure 5). Sera from vaccinated Pro+Vac or Vac pigs, which were highly protected against VirHRV challenge, did not have detectable amounts of IFNα at any post-challenge days. Moreover, serum IFNα levels of control (Pro and Control) pigs positively correlated (R=0.80; p=0.0431) with VirHRV shedding titers, and negatively correlated with frequencies of TLR3 expressing intestinal MNCs (significant only for duodenum: R=-0.67; p=0.039), suggesting that higher TLR3 expression levels in Pro pigs facilitated more efficient recognition of RV dsRNA and/or protection against HRV shedding.

### Frequencies of apoptotic MNCs were differentially affected by LGG+Bb12 colonization pre- and post-challenge

**Figure 6:**
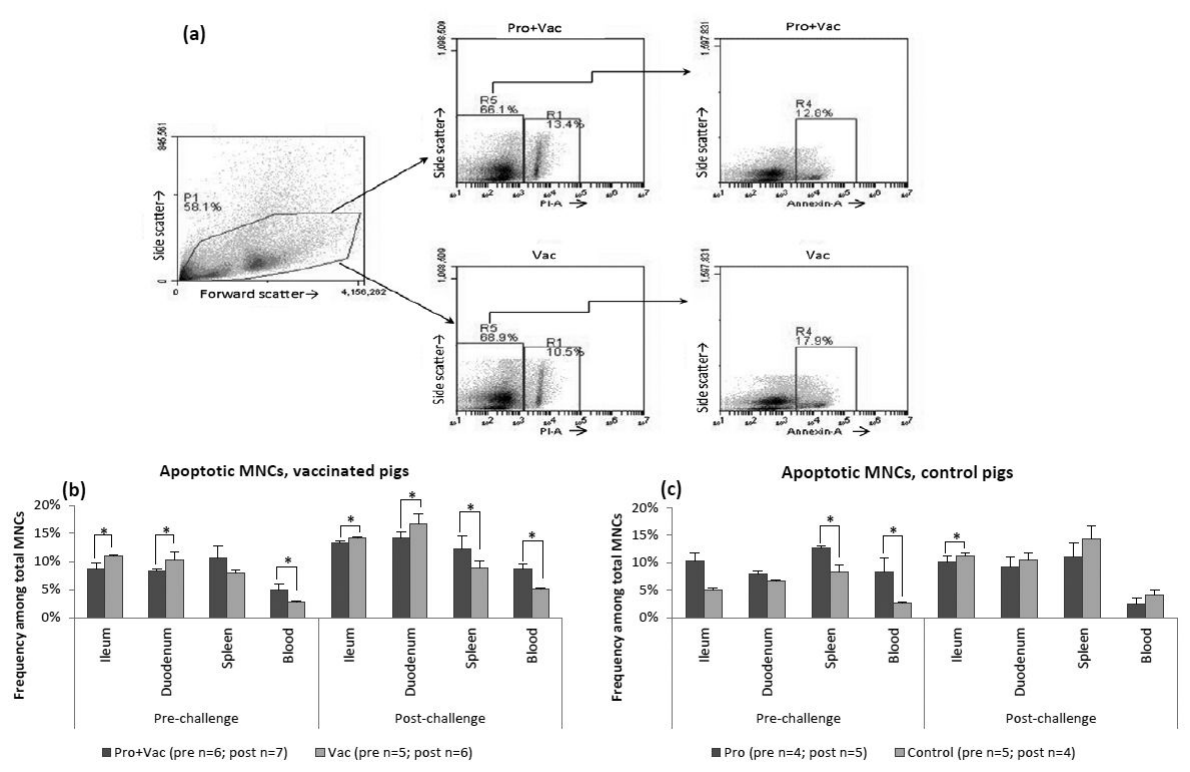
LGG+Bb12 colonization effect on frequencies and tissue distribution of apoptotic MNCs. Representative dot plot (**a**) demonstrates sequential gating strategy to detect apoptotic MNCs among total MNCs (for ileum samples from Pro+Vac and Vac pigs, post-challenge). Apoptotic MNC frequencies [assessed by Annexin V and Propidium Iodide staining (eBiosciences) and FACs analysis] pre- and post-challenge in gut (duodenum and ileum) and systemic tissues (spleen and blood) of (**b**) vaccinated and (**c**) control Gn pigs.

In this study, a consistent trend was that probiotic colonization decreased frequencies of apoptotic MNCs in duodenum and ileum (intestinal) and increased those in spleen and blood (systemic) of AttHRV vaccinated (Pro+Vac vs. Vac) pigs pre- and post-challenge (Figure 6b, Table 2). However, in unvaccinated control (Pro vs. Control) pigs, probiotic colonization resulted in increased frequencies of apoptotic MNCs both intestinally and systemically (Figure 6c, Table 2) pre-challenge; but apoptotic MNCs were lower in Pro compared to Control pigs post-challenge. Collectively, our results are indicative of a direct local (intestinal) cytoprotective effect of probiotics in the presence of AttHRV or VirHRV; while systemically these results demonstrate anti-proliferative effects of probiotics and their multifaceted interactions with AttHRV vaccine or VirHRV (Table 2). Additionally, apoptotic and TLR2 expressing MNC frequencies correlated significantly for ileum and duodenum (with R=0.778, p=0.022 and R=0.893, p=0.015, respectively) pre-challenge suggesting that TLR2 signaling may be involved in the induction of apoptosis in intestinal MNCs. Additionally, for vaccinated (Pro+Vac and Vac groups) and Control pigs, we observed moderately but significantly (p=0.0249) increased frequencies of apoptosis among systemic (1.17 fold increase for spleen and 1.08 fold increase for blood) and intestinal (1.4 fold increase for ileum and 1.53 fold increase for duodenum) MNCs post-challenge (Figure 6b,c). Probiotic colonized control pigs (Pro) did not have increased apoptotic MNC frequencies post-challenge as compared to pre-challenge, probably due to pro-apoptotic effects of LGG+Bb12 alone in non-vaccinated non-challenged pigs. Frequencies of necrotic MNCs post-challenge were significantly increased in duodenum of non-vaccinated pigs compared to their frequencies pre-challenge and significantly lower in all tissues of Pro+Vac and Vac pigs as compared to Pro and Control (data not shown).

**Table 2:** Effect of probiotic colonization on the frequencies of apoptotic and proliferating MNCs assessed by flow cytometry pre- and post-challenge, PID28/PCD0 and PID35/PCD7, respectively.

**AttHRV**	**VirHRV**	**Tissues**	**Group (VirHRV challenge status)**	**Apoptosis**	**Proliferation**
no	no	Intestinal (duodenum and ileum)	Pro (pre-challenge)	Increase	n/d
no	yes		Pro (post-challenge)	Decrease	Decrease
yes	no		Pro+Vac (pre-challenge)	Decrease	n/d
yes	yes		Pro+Vac (post-challenge)	Decrease	Decrease
no	no	Systemic (spleen and blood)	Pro (pre-challenge)	Increase	n/d
no	yes		Pro (post-challenge)	Decrease	Decrease
yes	no		Pro+Vac (pre-challenge)	Increase	n/d
yes	yes		Pro+Vac (post-challenge)	Increase	Decrease

Values greater or less than 1 correspond to increase or decrease of apoptotic or proliferating MNC frequencies, respectively. n/d – non-detectable.

### LGG+Bb12 colonization significantly decreased MNC proliferation ex vivo in VirHRV challenged pigs

**Figure 7:**
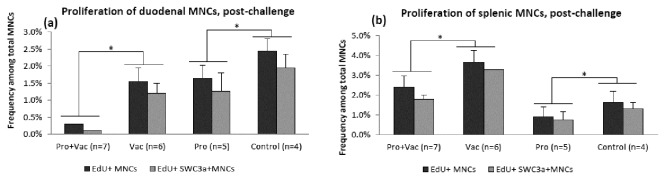
LGG+Bb12 colonization effect on MNC proliferation assessed *ex vivo*. (**a**) duodenal and (**b**) splenic MNCs and SWC3a+ MNCs (MNCs of myeloid origin) post-challenge assessed by FACs using Click-iT® EdU Alexa Fluor® 488 Flow Cytometry Assay Kit (Invitrogen). Asterisks indicate that non-colonized (Vac and Control) pigs had significantly (p<0.05) higher MNC and SWC3a+ MNC proliferation rates as compared to the colonized (Pro+Vac and Pro) pigs.

We assessed LGG+Bb12 colonization effects on basal MNC proliferation *ex vivo*. Similar to the findings by Shirkey et al. [12], we observed that the rates of MNC proliferation were highest in the proximal small intestine (duodenum) (data not shown); thus we were only able to measure the probiotics effects on basal *ex vivo* proliferation for duodenal (Figure 7a) and splenic (Figure 7b), but not for ileal MNCs.

Post VirHRV challenge, we observed a significant decrease in proliferation rates of duodenal and splenic total MNCs and MNCs of myeloid lineage (SWC3a+) in Pro+Vac and Pro groups compared to non-colonized pigs (Figure 7a, Table 2). Additionally, AttHRV vaccination resulted in decreased proliferation rates of duodenal MNCs, but increased proliferation rates of splenic MNCs post-challenge (Figure 7a,b). Interestingly, we did not observe probiotic effects on MNC proliferation in any treatment group pre-challenge (data not shown) probably due to very low levels of basal proliferation without VirHRV challenge. This suggests that post-challenge, probiotic colonization down-regulated VirHRV-induced MNC proliferation.

### LGG+Bb12 antagonized HRV-induced responses by splenic MNCs in vitro

**Figure 8:**
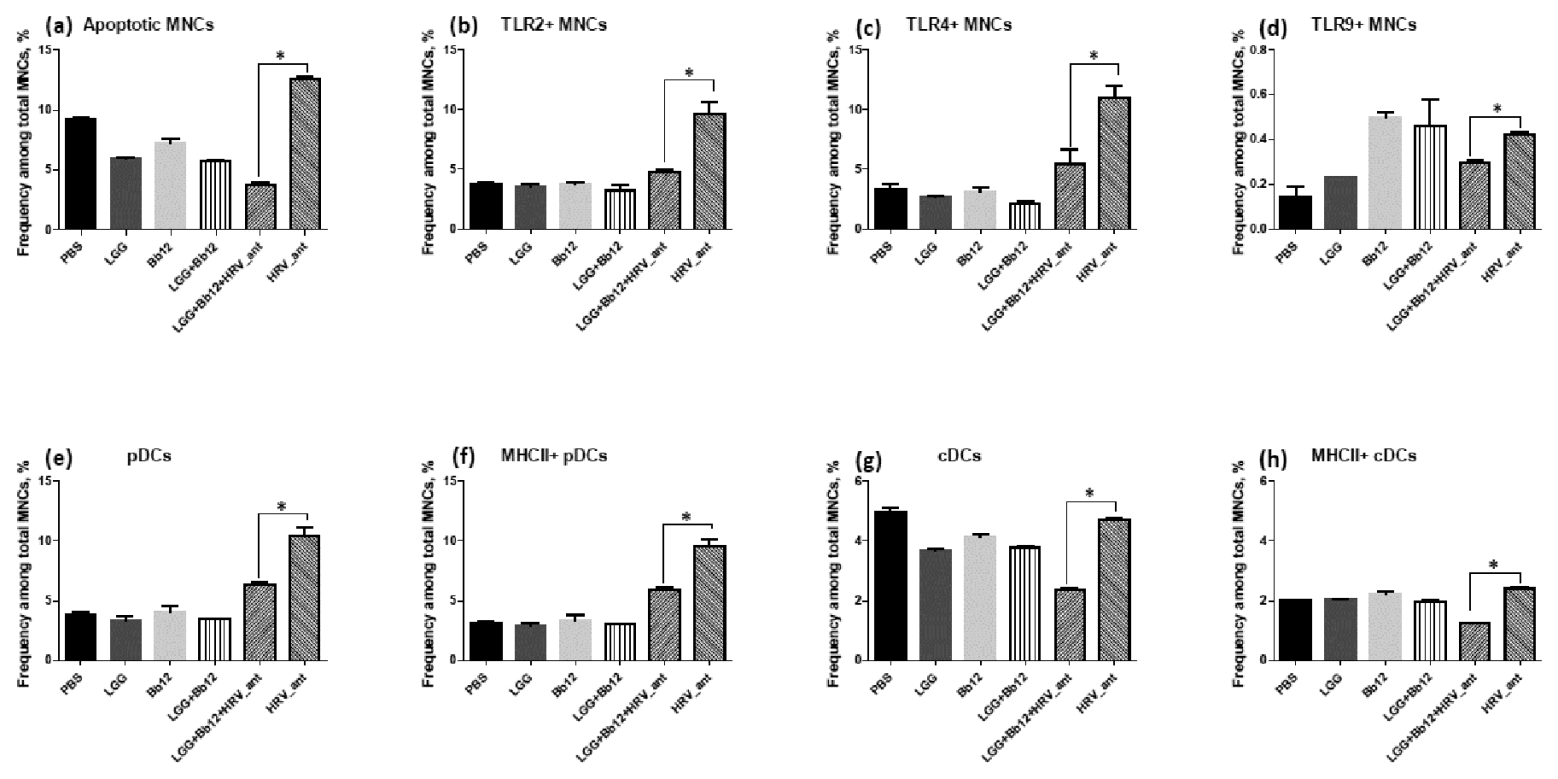
*In vitro* effects of stimulation with LGG, Bb12, LGG+Bb12, HRV antigen (HRV_ant) and LGG+Bb12+HRV_ant of splenic MNC [from control (not treated with AttHRV, VirHRV or probiotic *in vivo*) pigs]. Frequencies of (**a**) apoptotic, (**b**) TLR2+, (**c**) TLR4+, (**d**) TLR9+ MNCs and (**e**) pDCs, (**f**) MHCII+ pDCs, (**g**) cDCs, and (**h**) MHCII+ cDCs. The data are from three independent experiments, using duplicate treatments per trial.

We co-cultured splenic MNCs with LGG, Bb12, LGG+Bb12, LGG+Bb12+HRV and HRV to confirm some trends for probiotic/HRV effects on TLR expression frequencies observed *in vivo*. The general trend we observed *in vitro* was that inactivated HRV potently induced innate immune responses by splenic MNCs and that LGG+Bb12 moderated these HRV effects following LGG+B12+HRV co-stimulation (Figure 8). This indicates that LGG+Bb12 modulated innate immune responses induced non-replicating (inactivated) HRV *in vitro*, like we observed for live VirHRV *in vivo*. Consistent with the lack of marked LGG+Bb12 colonization effects on the innate immune responses pre-challenge *in vivo*, LGG and Bb12 treatment without HRV had low or no effect on the innate immune parameters tested. However, LGG+Bb12 significantly decreased frequencies of apoptotic, TLR2+, TLR4+ and TLR9+ MNCs and pDCs/cDCs induced by HRV. Unlike *in vivo*, LGG+Bb12 decreased basal levels of apoptosis among splenic MNCs. This suggests that spontaneous apoptosis *in vitro* naturally occurs at higher levels (than *in vivo*) during prolonged co-culture, shifting the balance between pro- and anti-apoptotic signaling by LGG+Bb12 towards a cytoprotective mode. LGG+Bb12 stimulation slightly decreased basal frequencies of TLR2+ and TLR4+ MNCs similar to what we observed *in vivo*. Interestingly, Bb12 or LGG+Bb12 induced the highest TLR9+ MNC frequencies during co-culture *in vitro*, but LGG+Bb12 decreased those induced by HRV. Additionally, pDC but not cDC frequencies were significantly increased by HRV stimulation; whereas LGG+Bb12 significantly decreased frequencies of both pDCs and cDCs when co-cultured with HRV. No TLR3 responses were evident probably due to lack of replicating HRV and resulting in insufficient dsRNA stimulation of splenic MNCs. Consistent with TLR and DC responses, cytokine production by the stimulated splenic MNCs revealed similar trends: inactivated HRV induced the highest levels of TGFβ, TNFα, IL10, IL12 and IFNα, which were decreased by LGG+Bb12+HRV co-stimulation (data not shown).

## Discussion

In humans, the complex microbiome and various underlying conditions complicate understanding of the interactions between commensals, pathogens and the immune system. Hence, Gn animals are indispensable for studying the effects of colonization with single or multiple bacterial species. Mono-associated pigs were previously shown to develop skewed immune and intestinal morphological responses similar to Gn animals [12]. We used dual lactobacilli-bifidobacteria (the two major phyla in naturally delivered and breastfed infants) colonization to achieve more balanced immune responses in a simplified system and to model direct interactions between HRV, commensal bacteria and the host. However, poor nutrition and hygiene (and associated infections) in developing countries may affect early development of the neonatal gut microbiome, possibly leading to the suppression and under-representation of these species [41-44]. This in turn may account for the increased RV infection severity and poor vaccine performance.

Probiotics modulate immunity by selective activation or suppression of various immune responses. The immune system of Gn mice [including gut-associated lymphoid tissue (GALT)] was shown to have decreased amounts of most cytokines, various T and B cell subsets, IgA, MHC class II, etc. Colonizing them with probiotics/commensals rescued most of these parameters [8]. Consistent with these findings, we observed increased frequencies of CD4, SWC3a, CD11R1 and MHCII expressing MNCs and cDCs in gut tissues and blood of colonized (Pro+Vac and Pro) pigs reflective of immunomaturation of Gn pigs. Interestingly, pDC and MHCII+ pDC frequencies were increased in ileum and blood but decreased in duodenum of colonized (Pro+Vac and Pro) as compared to non-colonized (Vac and Control) pigs. Human VirHRV Wa primarily replicates in villous epithelium of jejunum and ileum, and to a lesser extent of duodenum [45]. Therefore the observed differences in frequencies of ileal and duodenal pDCs (and other immune cells) may be related to the variations in VirHRV and LGG+Bb12 (also lowest in duodenum) loads in these sections of the small intestine. Noteworthy, decreased frequencies of pDCs (natural IFNα producers) in spleen and duodenum (but not in blood and ileum) of Pro as compared to Control pigs coincided with lower levels of IFNα in sera of Pro pigs post-challenge. Frequencies of CD4, CD11R1, SWC3a and MHCII expressing MNCs and DC subsets were decreased (mostly), unaffected or only minimally increased in spleen of Pro+Vac and Pro pigs as compared to Vac and Control pigs, likely representing an immunoregulatory effect of LGG+Bb12 colonization at the systemic level. These data are in agreement with our previous observations that LAB colonization significantly down-regulated HRV- infection-induced monocyte/macrophage activation/recruitment in the systemic lymphoid tissues (spleen) [27]. Different trends in inductive (Peyer’s patches of ileum) vs. effector (duodenum) tissues for pDCs/MNCs and similar trends for blood and ileum likely reflect the established MNC/DC mucosal trafficking patterns. Additionally, evidence from rodents and pigs suggests that active immune responses are initiated in the inductive intestinal site (ileum) and are likely controlled at the effector site (duodenum), with the latter one likely playing an immunoregulatory role [46]. It is consistent with our observations of more pronounced probiotic-induced immunoactivation in ileum than in duodenum.

In addition to major ligands (PGN and lipoproteins for TLR2 and LPS for TLR4), TLR2 and TLR4 recognize numerous exogenous and endogenous non-microbial ligands, including danger-associated molecular patterns and products of damaged tissue (heat shock proteins, endoplasmin, etc.) [47] that can result from HRV-induced apoptosis and initiate pro-inflammatory signaling.

Pre-challenge, TLR2+ (specific ligands present in LGG+Bb12) MNC frequencies were increased while TLR4+ (no defined ligand present) MNC frequencies were decreased in Pro vs. Control pigs demonstrating that the effect of LGG+Bb12 colonization alone (no AttHRV vaccine or VirHRV) was similar to previous findings in mice using *L. crispatus* [48]. Higher TLR2 expressing MNC frequencies without AttHRV vaccine or VirHRV challenge in colonized, non-vaccinated and non-challenged pigs (Pro group) may indicate a higher level of mucosal sensitivity to commensal gram-positive lactobacilli and bifidobacteria as compared to non-colonized Control pigs. This subsequently may contribute to Th2 cytokine release via TLR2 signaling as shown previously, and to dendritic cell maturation as we observed in this study and reported previously [49]. On the other hand, the down-regulation of TLR4 expression that is often involved in pro-inflammatory signaling may prevent the release of an excess of pro-inflammatory cytokines and inhibit epithelial cell migration, thereby regulating gut homeostasis [50]. Frequencies of TLR2 and TLR4 expressing MNCs were decreased pre-challenge in LGG+Bb12 colonized vaccinated (Pro+Vac) pigs as compared to non-colonized (Vac) pigs likely counteracting AttHRV induced TLR2 expression.

Bacterial DNA or viral ssRNA (present during replication of dsRNA viruses, including RV) containing unmethylated CpG motifs are potent ligands for TLR9 [51]. Therefore, it is not unexpected that we observed a synergistic interaction between AttHRV and LGG+Bb12 that resulted in increased frequencies of TLR9 expressing MNCs in gut tissues of Pro+Vac pigs compared to all other groups pre-challenge. Increased frequencies of TLR9+ MNCs pre-challenge coincided with increased levels of protection against VirHRV diarrhea and shedding in this group post-challenge. These observations are consistent with previous reports on anti-inflammatory signaling via TLR9 that resulted in attenuation of experimental colitis or *Helicobacter pylori*-induced gastritis in mice [52,53].

TLR3 is involved in the initial recognition of RV genomic dsRNA. Thus, vaccinated (pre-exposed to AttHRV) animals (Pro+Vac and Vac) were largely protected from VirHRV replication post-challenge and had undetectable levels of TLR3 expression by MNCs. Increased frequencies of TLR3 expressing MNCs from colonized control (Pro) compared to Control pigs indicates that probiotic colonization may have either assisted in virus-host interactions and immunoactivation of MNCs or improved survival of TLR3+ MNCs. Our findings are in agreement with the recent data showing that mainly TLR3-mediated immune responses restrict RV replication in adults compared to immature TLR3 responses and increased susceptibility to RV in neonates [54]. Additionally, the negative correlation between TLR3 expressing MNC frequencies (higher in Pro pigs) and the IFNα responses (higher in Control pigs) suggests that TLR3 pathway activation is critical for anti-HRV responses, whereas HRV-induced IFNα levels are not a correlate of protection in our Gn pig model. Overall, our results suggest that a balance between immunoactivation and immunomodulation in the context of HRV vaccine and infection is likely achieved by probiotics through differential regulation of TLR signaling in Gn pigs.

Rotavirus was shown to increase apoptosis and proliferation of intestinal epithelial cells in a mouse model increasing an overall epithelial cell turnover *in vivo* [37]. Consistent with that, we observed increased frequencies of apoptotic MNCs in duodenum and ileum in Pro+Vac, Vac and Control pigs post-VirHRV challenge (vs. pre-challenge). As evident pre-challenge, LGG+Bb12 colonization increased apoptotic MNC frequencies intestinally and systemically in Pro compared to Control pigs, which is consistent with the recognized anti-proliferative action of probiotics and LGG in particular [55]. However, post-challenge LGG+Bb12 colonization resulted in decreased frequencies of apoptotic MNCs (in Pro vs. Control pigs) possibly through providing partial protection from VirHRV replication and therefore decreasing VirHRV-induced cytokine-mediated apoptosis. Noteworthy, in vaccinated pigs LGG+Bb12 colonization resulted in decreased apoptotic MNC frequencies (in Pro+Vac vs. Vac pigs) in the intestine but increased those systemically pre- and post-challenge. This is in agreement with the fact that AttHRV vaccination resulted in significantly decreased MNC proliferation rates intestinally but increased those systemically. Additionally, LGG+Bb12 colonization decreased MNC proliferation rates post-challenge contributing to decreased MNC cell turnover. Overall, LGG+Bb12 colonization evidently antagonized AttHRV/VirHRV effects on cell survival and proliferation.

Finally, our *in vitro* results indicate that modulatory effects of LGG+Bb12 on the immune responses to AttHRV vaccine or VirHRV *in vivo* cannot be simply explained by probiotic colonization of the intestine and subsequent decrease of HRV replication due to direct commensal-pathogen interactions. Rather these effects are achieved via differential TLR signaling and modulation of other innate immune parameters that can result in decreased VirHRV replication. Additionally, our results confirm that not only TLR3, as we observed *in vivo*, but also TLR2, TLR4 and TLR9 responses were induced/modulated by HRV as we demonstrated *in vitro* and as reported previously [40].

Thus, we demonstrated that probiotic bacteria colonization in the neonatal period is critical for immunomaturation, immune homeostasis and may aid in protection against enteric viral pathogens. Our results indicate that colonization with LGG+Bb12 moderated VirHRV infection in the neonatal Gn pig disease model. Additionally, LGG+Bb12 acted as an immunostimulant for AttHRV vaccine via differential TLR signaling that modulated DC activation and responses. Such information is of importance for development and implementation of practical, safe, and economic interventions and possibly preventive tools to moderate childhood diarrhea and to reduce the associated mortality in developing countries.

## Supporting Information

Table S1 **Antibodies used for flow cytometry analyses.**
(DOC)
