# Supplementary material for: Lactobacilli and Bifidobacteria Promote Immune Homeostasis by Modulating Innate Immune Responses to Human Rotavirus in Neonatal Gnotobiotic Pigs
Source: PLoS One. 2013 Oct 2;8(10):e76962. doi: 10.1371/journal.pone.0076962 (PMC3788735; doi:10.1371/journal.pone.0076962)
Supplement: Table S1 — Antibodies used for flow cytometry analyses. (DOC) [file pone.0076962.s001.doc]

**Table S1. Anti**bodies used for flow cytometry analyses.

| **Marker** | **Fluorochrome** | **Antibody/Vendor/Cat. #** | **Isotype** | **Note** |
| --- | --- | --- | --- | --- |
| SWC3a (porcine) | PE | Mouse Anti-porcine Monocyte/Granulocyte-PE/Southern Biotech/ 4525-09 | IgG1 | Human analog CD172 |
| CD4 (porcine) | SPRD | Mouse Anti-porcine CD4a/Southern Biotech/4515-13 | IgG2b |  |
| CD11R1 (porcine) | No | Mouse Anti-pig CD11R1/Serotec/MCA1220 | IgG1 | Human analog CD11b |
| TLR2 (human) | PE | PE-Anti-Human TLR2, eBioscience, clone T2.5 | IgG1 | Cross reacts with porcine TLR2 |
| TLR3 (human) | PE | PE-Anti-Human TLR3/eBiosciences/clone: TLR3.7/12-9039-82 | IgG1 | Cross reacts with porcine TLR3 |
| TLR4 (human) | PE | PE-Anti-Human TLR4/eBiosciences/clone: HTA125/12-9917-41 | IgG2a | Cross reacts with porcine TLR4 |
| TLR9 (human) | PE | PE-Anti-Human TLR9/eBiosciences/clone: eB72-1665 | rat IgG2a | Cross reacts with porcine TLR9 |
| Isotype control, IgG1-PE | PE | Mouse IgG1-PE/Southern Biotech/0102-09 | IgG1 | Isotype control SWC3a PE |
| Isotype control, IgG2b-SPRD | SPRD | Mouse IgG2b-SPRD/Southern Biotech/0104-13 | IgG2b | Isotype control CD4 SPRD |
| Isotype control, IgG1-FITC | FITC | Mouse IgG1 K Isotype Control FITC/eBiosciences/11-4714-42 | IgG1 | Isotype control CD103 FITC |
| Secondary antibody, anti-mouse IgG1, APC | APC | APC Rat Anti-Mouse IgG1, clone X56/BD biosciences/550874 | IgG1 | Secondary antibody for CD11R1 |
